# The development of the effect of peer monitoring on generosity differs among elementary school-age boys and girls

**DOI:** 10.3389/fpsyg.2015.00895

**Published:** 2015-06-29

**Authors:** Haruto Takagishi, Takayuki Fujii, Michiko Koizumi, Joanna Schug, Fumihiko Nakamura, Shinya Kameshima

**Affiliations:** ^1^Graduate School of Brain Sciences, Tamagawa University, Machida, Tokyo, Japan; ^2^Research Center for Child Mental Development, University of Fukui, Fukui, Japan; ^3^Department of Psychology, College of William and Mary, Williamsburg, VA, USA; ^4^Tsuda Hospital, Hirakata, Japan; ^5^Department of Social Welfare, Kansai University of Welfare Sciences, Kashiwara, Japan

**Keywords:** generosity, observer effects, economic game, children, sex difference

## Abstract

The purpose of this study was to examine the effect of peer monitoring on generosity in boys and girls aged 6–12 years. A total of 120 elementary school students played a one-shot dictator game (DG) with and without peer monitoring by classmates. Children decided how to divide 10 chocolates between themselves and a classmate either in a condition in which their allocations were visible to their peers, or in private. While the effect of peer monitoring on the allocation amount in the DG was clearly present in boys, it was not observed in girls. Furthermore, the effect of peer monitoring in boys appeared at the age of 9 years. These results suggest that the motivation to draw peers’ attention plays a stronger role for older boys than for girls or younger boys. The potential roles of higher-order theory of mind, social roles, and emergence of secondary sex characteristics on the influence of peer monitoring on generosity shown by boys are discussed.

## Introduction

People are highly motivated to build and maintain a good reputation, paying a great deal of attention to social evaluations and keeping these evaluations positive in nature (Fehr, 2004; Bénabou and Tirole, 2006; Izuma et al., 2010; Izuma, 2012). Consistent with these findings, a number of studies have shown that people tend to behave in a more generous and pro-social manner when they are being observed by others. This tendency occurs not only in adults (Bereczkei et al., 2007; Iredale et al., 2008; Izuma et al., 2010; Van Vugt and Iredale, 2013) but also in children (Engelmann et al., 2012, 2013; Houser et al., 2012; Leimgruber et al., 2012; Fujii et al., 2015). The tendency for people to behave in a more pro-social manner when they are observed by others, and thus may accrue reputation, is so strong that it can be elicited even by subtle cues of monitoring such as pictures of eyes (Haley and Fessler, 2005; Bateson et al., 2006; Mifune et al., 2010; Keller and Pfattheicher, 2011; Oda et al., 2011; Ekström, 2012; Nettle et al., 2013) and even three dots in a “watching eyes” configuration (Rigdon et al., 2009). Sensitivity to monitoring by others is thought to be a psychological mechanism evolved to help humans adapt to social environments where reputation is extremely important in acquiring resources (Milinski et al., 2002).

The evolutionary basis for sensitivity to peer monitoring has been explained by two theories: *indirect reciprocity* (e.g., Alexander, 1987; Nowak and Sigmund, 1998) and *signaling theory* (e.g., Zahavi, 1975; Roberts, 1998). According to the perspective of indirect reciprocity, people are thought to have a strong motivation to build a good reputation in social exchange domains in order to elicit favors from third parties, thus people become sensitive to monitoring by others. Consistent with this notion, studies have shown that people tend to base their behaviors on how others have behaved in the past (Wedekind and Milinski, 2000) and that generous people receive more assistance from third-parties (Kato-Shimizu et al., 2013). Signaling theory proposes that people can use costly signals, such as by giving generous allocations to others, to honestly signal their cooperative nature. One instance of signaling theory is the handicap principle, which proposes that for males (who typically are tasked with demonstrating their fitness to females) generosity to others represents a signal of quality in the mate choice domain (Zahavi, 1975; Roberts, 1998). That is, showing generosity to others is a costly signal of mate quality, and enhancing reputation in this domain will allow more access to reproductive opportunities for males in particular. While the former theory predicts the effect of peer monitoring on generosity in both men and women, the latter theory predicts sex differences.

Although a number of studies have demonstrated the development of generosity in children (Benenson et al., 2007; Fehr et al., 2008; Gummerum et al., 2008), none have examined the effect of peer monitoring. Thus, in this study we sought to examine the emergence of sensitivity to peer monitoring in elementary school-age children. We specifically sought to examine whether peer monitoring effects would emerge around the age of 9–10 years of age, an age group associated with the development of higher order theory of mind (ToM), thought to be the cognitive basis for representing how others think about one’s self (Amodio and Frith, 2006; Frith and Frith, 2008), and is thus a requirement for attention to monitoring by peers.

We also sought to examine potential sex differences in the development of sensitivity to peer monitoring among older elementary school-age children. Although five studies have examined the effect of peer monitoring in 5-year-old children, none of these studies have reported sex differences (Engelmann et al., 2012, 2013; Leimgruber et al., 2012; Fujii et al., 2015). However, several studies of adults have reported sex differences (Iredale et al., 2008; Rigdon et al., 2009; Van Vugt and Iredale, 2013). For example, Rigdon et al. (2009) showed that the effect of three dots in a watching eye configuration, proposed to suggest monitoring from others, on generosity in a dictator game (DG) was observed only in male participants. Furthermore, several studies have shown that men tend to give more money to another person in the DG (Iredale et al., 2008) and contribute more to the group in the public goods game (Van Vugt and Iredale, 2013), particularly when observed by women. More recently, Raihani and Smith (2015) showed that men were more likely to increase donations to fundraisers when donating to attractive female volunteers, particularly in response to large donations made by other men. Together, these results indicate that adult men are highly sensitive to monitoring and may try to appeal to others (particularly to women) by demonstrating their generosity when they are monitored by others.

Thus, in this study we examine in elementary school-age boys and girls in order to examine whether sensitivity to peer monitoring develops during this developmental period, which coincides with the emergence of higher order ToM and the onset of puberty. We also sought to examine sex differences to examine whether sensitivity to peer monitoring in adult men shown in prior studies (e.g., Iredale et al., 2008; Rigdon et al., 2009; Van Vugt and Iredale, 2013; Raihani and Smith, 2015) also emerges at this developmental period. To do so, we used a simple economic game known as the dictator game (Kahneman et al., 1986), widely used in other developmental literature (Benenson et al., 2007; Fehr et al., 2008; Gummerum et al., 2008; Engelmann et al., 2013; Fujii et al., 2015), to measure generosity in children.

## Materials and Methods

### Ethics Statement

The ethical committee at the Center for Experimental Research in Social Sciences, Hokkaido University approved this study and consent was obtained from all the students and teacher at the elementary school. The methods were carried out in accordance with the approved guidelines.

### Participants

A total of 120 children (60 girls and 60 boys; mean age = 9.2, SD = 1.5) attending a private elementary school in Japan participated in this study. Participants were recruited from five grades: second grade (12 girls and 12 boys; mean age = 7.1 years, SD = 0.3), third grade (12 girls and 12 boys; mean age = 8.3 years, SD = 0.4), fourth grade (12 girls and 12 boys; mean age = 9.4 years, SD = 0.5), fifth grade (12 girls and 12 boys; mean age = 10.2 years, SD = 0.4), and sixth grade (12 girls and 12 boys; mean age = 11.2 years, SD = 0.4).

### Dictator Game

**FIGURE 1:**
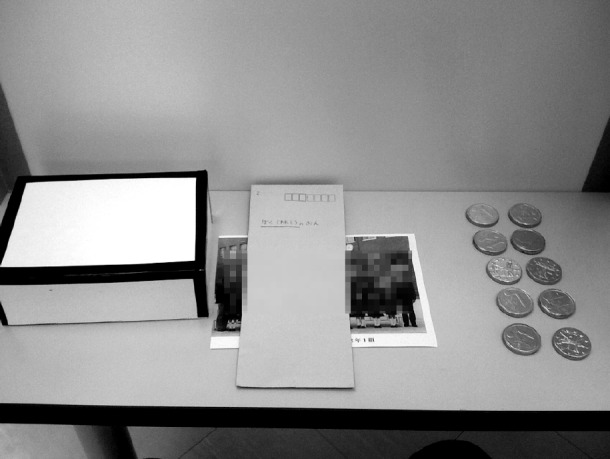
**Photograph of the top of a desk in the private condition (left, box with lid for the recipients’ chocolates; center, envelope for the child’s own chocolates and image of classmates; right, 10 chocolates)**.

All participants played a one-shot DG. In each session, 12 children from the same grade and class but of mixed sex (six boys and six girls) played the game in a quiet room. Only a male experimenter and 12 children were in the room, and the room was closed such that individuals not involved in the study could not observe participants. At the beginning of the experiment, the 12 children sat on chairs at their desks arranged in a circle and were told the rules of the DG by the experimenter, who stood at the center of the desks. After describing of the game, the experimenter orally confirmed participants’ understanding of the rules of the DG. Then each child received 10 coin chocolates from the experimenter and was tasked with deciding how to divide these chocolates between him/herself and a recipient. A box, an envelope, and a photograph of all classmates were placed on each desk (Figure 1). All children were told that one of the classmates in the photo would be randomly assigned the role of the recipient of the allocated chocolates after the experiment. To remove potential expectations of reciprocity, participants were informed that they would not be told which child received the allocated chocolates. Children were told that, if they wanted to give the chocolates to a recipient, they should put the chocolates into the box, but if they wanted to keep the chocolates for themselves, that they should put them into the envelope.

**FIGURE 2:**
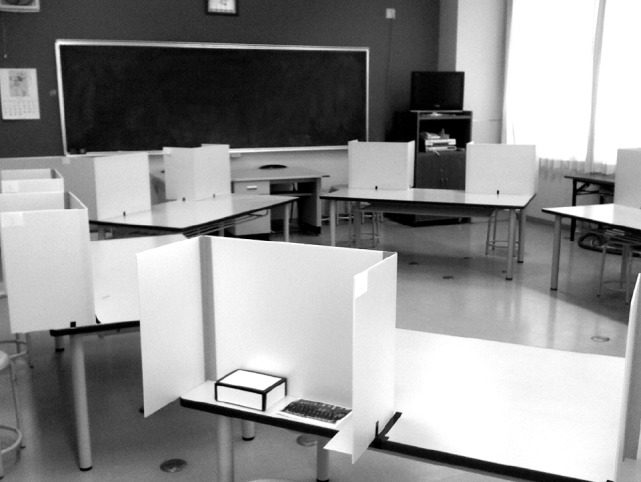
**Photograph of the experimental environment in the private condition. In the public condition, the vertical plastic boards and the lids of the boxes were removed**.

Half of the participants played the DG in the private condition, and the remaining participants played the DG in the public condition, resulting in five sessions for each condition. Each condition was conducted alternately. In the private condition, vertical, light orange plastic boards obscured each desk and a lid covered the box, and the experimenter told participants not to look at what the other children were doing. Thus, the children could not see how many chocolates their peers put into the box (Figure 2). In the public condition, the plastic boards and the lids were absent and the children were able to observe the number of chocolates that the other children put into the box. The experimenter stood in the center of the room during the entire experiment, and thus he could not see the amount of the chocolates in the box in the private condition. All children made their decisions at the same time, and signaled to the experimenter by raising their hand when they had completed the task, at which point the experimenter collected the boxes. After the all children had finished their decision, they took the envelopes and left the room.

## Results

**FIGURE 3:**
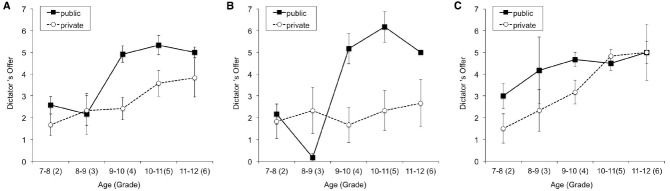
**Mean level of dictator’s offer in each condition. All children (A), boys (B), and girls (C).** Error bars indicate standard error of the mean.

The mean allocations by sex and grade in each condition are presented in Figure 3. A 5 × 2 × 2 Grade [(2, 3, 4, 5, and 6) × Sex (girl = 0, boy = 1)] × Condition (private = 0, public = 1) three-way analysis of variance (ANOVA) on the allocations revealed significant main effects of grade, *F*(4, 100) = 8.44, *p* < 0.001, ${\text{η}}_{p}^{2}$ = 0.252, condition, *F*(1, 100) = 12.41, *p* = 0.001, ${\text{η}}_{p}^{2}$ = 0.11, sex, *F*(1, 100) = 6.13, *p* = 0.015, ${\text{η}}_{p}^{2}$ = 0.058, and a significant three-way interaction (grade × sex × condition), *F*(4, 100) = 4.28, *p* = 0.003, ${\text{η}}_{p}^{2}$ = 0.146.

Based on the significant interaction, a 5 × 2 [Grade (2, 3, 4, 5, and 6) × Condition (private = 0, public = 1)] two-way ANOVA was conducted separately for each sex. In boys, significant main effects of grade, *F*(4, 50) = 5.74, *p* = 0.001, ${\text{η}}_{p}^{2}$ = 0.315, and condition, *F* (1, 50) = 10.86, *p* = 0.002, ${\text{η}}_{p}^{2}$ = 0.178, as well as a significant interaction effect, *F*(4, 50) = 5.51, *p* = 0.001, ${\text{η}}_{p}^{2}$ = 0.306, were observed (Figure 3B). A *post hoc* test revealed that the allocation amount in the public condition was significantly higher than it was in the private condition from the fourth to the sixth grade after the Bonferroni correction (fourth grade: *p* = 0.002, fifth grade: *p* = 0.001, sixth grade: *p* = 0.033). For girls, a significant effect of grade was observed, *F*(4, 50) = 3.72, *p* = 0.010, ${\text{η}}_{p}^{2}$ = 0.229, while the effect of condition, *F*(1, 50) = 3.07, *p* = 0.086, ${\text{η}}_{p}^{2}$ = 0.058, and the interaction, *F*(4, 50) = 0.74, *p* = 0.568, ${\text{η}}_{p}^{2}$ = 0.056, did not reach significance (Figure 3C).

## Discussion

In this study, we examined the effect of peer monitoring on generosity in elementary school-aged children, and observed three major findings. First, the amount of the allocation in the DG increased with age in both boys and girls, consistent with previous studies (Benenson et al., 2007; Fehr et al., 2008). Second, the effect of peer monitoring was observed only in boys who were at least 9–10 years of age (i.e., forth to sixth grade). While the amount of the dictator’s offer to the recipient in the public condition increased with age in boys, it remained low in the private condition. Third, in girls, the amount of the dictator’s offer to the recipient in both conditions increased with age, and an effect of monitoring was not observed. These results indicate that peer monitoring enhances generosity in boys but not in girls, and further, that older boys are more sensitive to monitoring by peers. This is the first study to show differences between boys and girls in the effect of peer monitoring on generosity using the DG.

There are a number of theoretical frameworks which may help to interpret differences in the development of the sensitivity to peer monitoring between boys and girls. For instance, social role theory (Hindin, 2007) may help to explain gender differences in children’s generosity. In social role theory, human behavior is thought to be strongly affected by social roles constructed by one’s culture, and studies stemming from this perspective have shown that men tend to be helpful in the public activities (Eagly and Crowley, 1986; Eagly, 1987), and that that this tendency strengthens in the presence of an audience (Eagly, 1987). While this theory does not account for age differences observed in this study, it is possible that older boys in our study might be more sensitive to social roles and were thus more generous to others.

Another factor which may explain the emergence of sensitivity to peer monitoring is the development of higher-order ToM, which is considered the cognitive basis for the representation of one’s own reputation in the eyes of others (Amodio and Frith, 2006; Frith and Frith, 2008). In order to understand one’s own reputation, it is necessary to consider what others think of oneself. Thus, sensitivity to peer monitoring requires the development of higher order ToM. Consistent with this interpretation, Houser et al. (2012) demonstrated a peer monitoring effect on contributions to group members using a modified social dilemma game in children 9 years and older. Although Houser et al. (2012) did not investigate sex differences in peer monitoring effects in children, our study clearly showed significant differences between boys and girls. This discrepancy may be explained by the different games; while the DG was used in our study to measure children’s generosity, a modified social dilemma game that involved waiting for a reward was used in Houser et al. (2012)’s study. The latter study did not measure children’s generosity *per se*, but rather their endurance (Curry et al., 2008) and general trust (Yamagishi, 1988).

While we cannot conclude that the development of sensitivity to peer monitoring in our study is due to the emergence of secondary sexual characteristics and in the older boys, this result is consistent with the handicap principle which predicts that boys are strongly motivated to display their generosity as a costly signal to appeal to girls. As testosterone secretion increases with the onset of secondary sexual characteristics and sexual maturity, secondary sexual characteristics in boys emerge around 9–10 years of age (Matsuo, 1993; Herman-Giddens et al., 2001, 2012), at which point testosterone increases, and boys start showing interest in the opposite sex (assuming a heterosexual orientation). Thus, if sex differences in the effect of peer monitoring on generosity are impacted by testosterone, these differences should theoretically appear around the age of 9–10 years of age.

The interpretation that increases in generosity shown by males in response to peer monitoring is related to secondary sexual characteristics is consistent with findings of several studies of adults, which have shown that generosity in the context of economic games is associated with male sex hormones (e.g., testosterone; Zak et al., 2009; Boksem et al., 2013) as well as masculinized facial structure (Stirrat and Perrett, 2012), thought to be an index of male hormone (e.g., testosterone) concentration. These studies provide some evidence to support the notion that generosity signals quality in mating domains, consistant with the handicap principle, particularly for men. While this study found the development of sensitivity to peer monitoring was consistent with the timing of the onset of secondary sexual characteristics in boys, further research is needed to measure testosterone concentration and to examine whether testosterone levels are associated with the increase in the effect of monitoring on generosity in boys.

Furthermore, other studies might examine whether the effects of this study are impacted by culture. Indeed, social role theory and sexual signaling theories are not necessarily mutually exclusive, as the manner in which humans signal quality in mating domains is highly dependent on culturally defined roles. For instance, in some cultures men might drive fast and dangerous cars, and in other cultures men might hunt dangerous game animals as a costly signal to appeal to potential mates. As an example, research on the “Culture of Honor” in the American South has suggested that higher levels of violence observed in white men in this region is associated with men’s (erroneous) perceptions of the extent to which women prefer men who react violently to threats to reputation (e.g., Vandello et al., 2009). Thus, cultural variation norms regarding acceptable gender roles can influence the types of costly signals relevant in mating domains.

Overall, this study found evidence suggesting that, not only does generosity in children in the DG increase with age, the effect of monitoring on generosity emerges in boys around 9 years of age. We hope this study will encourage future research examining contributions of potential mechanisms, such as male sex hormones, social roles, or the development of higher order theory of mind, which may account for these results.

## Author Contributions

Conceived and designed the experiments: HT SK JS. Performed the experiments: HT FN SK. Analyzed the data: HT TF MK. Contributed reagents/materials/analysis tools: HT FN SK. Contributed to the writing of the manuscript: HT TF MK JS.

### Conflict of Interest Statement

The authors declare that the research was conducted in the absence of any commercial or financial relationships that could be construed as a potential conflict of interest.
